# Colocalization Analysis of Cytoplasmic Actin Isoforms Distribution in Endothelial Cells

**DOI:** 10.3390/biomedicines10123194

**Published:** 2022-12-09

**Authors:** Anton S. Shakhov, Polina A. Kovaleva, Alexandra S. Churkina, Igor I. Kireev, Irina B. Alieva

**Affiliations:** A.N. Belozersky Institute of Physical and Chemical Biology, Lomonosov Moscow State University, 119992 Moscow, Russia

**Keywords:** endothelial cell, actin cytoskeleton, non-muscle actin isoforms, β-actin, γ-actin, super resolution microscopy, colocalization analysis

## Abstract

Actin cytoskeleton is an essential component of living cells and plays a decisive role in many cellular processes. In mammals, β- and γ-actin are cytoplasmic actin isoforms in non-muscle cells. Despite minor differences in the amino acid sequence, β- and γ-actin localize in different cell structures and perform different functions. While cytoplasmic β-actin is involved in many intracellular processes including cell contraction, γ-actin is responsible for cell mobility and promotes tumor transformation. Numerous studies demonstrate that β- and γ-actin are spatially separated in the cytoplasm of fibroblasts and epithelial cells; this separation is functionally determined. The spatial location of β/γ-actin in endothelial cells is still a subject for discussion. Using super-resolution microscopy, we investigated the β/γ-actin colocalization in endotheliocytes and showed that the β/γ-actin colocalization degree varies widely between different parts of the marginal regions and near the cell nucleus. In the basal cytoplasm, β-actin predominates, while the ratio of isoforms evens out as it moves to the apical cytoplasm. Thus, our colocalization analysis suggests that β- and γ-actin are segregated in the endotheliocyte cytoplasm. The segregation is greatly enhanced during cell lamella activation in the nocodazole-induced endothelial barrier dysfunction, reflecting a different functional role of cytoplasmic actin isoforms in endothelial cells.

## 1. Introduction

Actin is one of the most abundant proteins in a living cell. Actin is highly dynamic and can polymerize in filaments; however, actin also forms other intracellular structures such as rounded microparticles in endotheliocytes [1,2,3,4]. Actin structures are found in all cells of a living organism and are involved in maintaining and changing the shape of cells, processes of exocytosis and endocytosis, adhesion of cells to substratum and cell movement, and signal transduction [5,6]. The number of actin genes varies significantly in different groups of organisms, but most mammals have six actin isoforms: four muscle (α-skeletal, α-cardiac, α- and γ-smooth muscle) and two non-muscle (β- and γ-cytoplasmic) [7]. Actin proteins are similar in amino acid sequences: the amino acid sequences of cytoplasmic β- and γ-actin differ only in four residues at the N-terminus [8]. In addition, isoforms have different isoelectric points [8]. Tissues differ greatly in the isoform expression level. Unlike tissue-specific actin isoforms, non-muscle β- and γ-actin are found in all cell types. Non-muscle actin isoforms interact with myosin motor proteins in a special way [9]: non-muscle myosin 2A, 2B, and 2C1 preferentially bind to β- and γ- but not to α-actin. Compared to α-actin, cytoplasmic actin isoforms are 4-fold more effective in the activation of myosin 2A or 2B ATPase [9]. Over the past decade, a number of differences have been reported in the organization of structures formed by cytoplasmic β- and γ-actin. By 2009, highly specific monoclonal antibodies were obtained selective to cytoplasmic β- or γ-actin [1]. This tool largely made it possible to advance our understanding of how β- and γ-actin are located in cells relative to each other and what functions they perform in various cell types. As it turned out, in fibroblasts and epithelial cells, two actin isoforms are spatially separated, [1]: β-actin is found mainly in stress fibers, circular bundles, and near the intercellular contacts; in contrast, γ-actin forms a network in the cortical areas and lamellipodia. This spatial separation reflects the functional differences of the isoforms. In the epithelial cells, β-actin forms basal microfilament bundles and participates in the adhesion junctions; γ-actin organizes the cortical (dorsal) network of actin filaments and some stress fibers.

Endothelial cells line the vessel inner surface and form tight contacts with each other to perform a specific barrier function. The actin cytoskeleton is indispensable for this function; the functional activity of actin structures is key for endothelium functions, as well as for normal and tumor angiogenesis. However, the spatial arrangement of two non-muscle actin isoforms in endothelial cells is still a subject for active discussion. Some authors suggest that β- and γ-actin are not spatially separated in thin and flat endothelial cells, i.e., the isoforms are colocalized [10]. Other authors suggest that β- and γ-actin are segregated in the cytoplasm; the latter view is consistent with differences in functional activity of β- and γ-actin [3,11,12,13]. This, in fact, adds fundamental value to the issue, provided that the activity of actin cytoskeleton determines normal endothelial barrier function. The disagreement may have arisen due to purely methodological issues. Most studies on the endothelial cell cytoskeletons were performed with the use of confocal microscopy, which gives good resolution in the XY but not *Z*-axis. Here, we aimed to analyze the degree of colocalization of the β- and γ-actin systems in various individual localities in the endothelial cell. We applied one of the super resolution microscopy methods, Structured Illumination Microscopy (SIM), which allowed us to reconstruct the entire cell volume with high resolution not only along the XY-axes but also along *Z*-axis.

## 2. Results and Discussion

**Figure 1:**
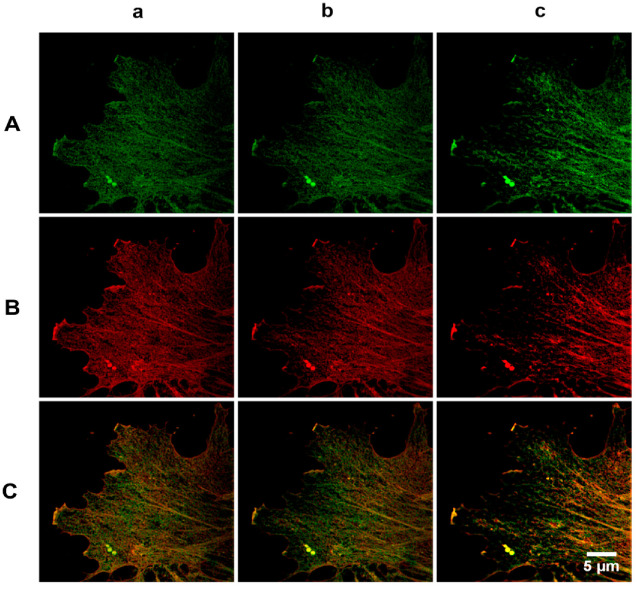
Cytoplasmic β- and γ-actin in human pulmonary artery endothelial cells (HPAEC). Horizontal panel (**A**), immunofluorescent staining of β-actin; horizontal panel (**B**), immunofluorescent staining of γ-actin; horizontal panel (**C**), merged. Three consecutive optical sections (three vertical panels) are presented: the section with the most clearly distinguishable structures (panel (**b**)) and two adjacent sections, above (panel (**a**)) and below (panel (**c**)) the selected one.

The main problem motivating this study is how β- and γ-actin are distributed in the cytoplasm of endothelial cells: whether the isoforms colocalize or there are differences in their localization in particular cytoplasm regions characterized by certain functional features (for example, in the lamella edge or by the intercellular contacts). For quantification of β- and γ-actin colocalization, we used immunostaining with highly specific monoclonal antibodies [1]. This approach allowed us to clearly differentiate the distribution of cytoplasmic β- and γ-actin isoforms in pulmonary artery (Figure 1) and vein endothelial cells.

### 2.1. Selection of a Correlation Analysis Method

Previously, using the same specific antibodies, other authors analyzed the mutual arrangement of β- and γ-actin structures in two endothelial cell cultures, HMEC-1 (microvascular endothelial cells) and BMH29L (bone marrow endothelium) [10]. The authors acquired fluorescent images with the use of confocal microscopy and applied the ImageJ software and the Coloc2 plugin for analyses of selected single cells. As a preliminary step, they subtracted the background and then chose a certain region of interest (ROI) excluding the dark areas surrounding the cell. Based on the Pearson coefficient (PCC) calculation over the entire cell area, the authors concluded that colocalization of β- and γ-actin was high [10]. Notably, the authors pointed out a number of cells falling out of the general row of high colocalization (PCC = 0.5 and below), which, in their opinion, is associated with the difference in stoichiometry.

Scatterplots of pixel intensity of cells that showed low correlation coefficients admit another interpretations [10], which motivated us to conduct a focused study. For the first time in a β- and γ-actin colocalization study, we used SIM microscopy, which gives good resolution along the *Z*-axis, while previous studies relied on images acquired with the use of confocal microscopy, which has lower *Z*-axis resolution [10,11,12,13,14,15,16,17].

Currently, the most widely used methods of correlation analysis are the Pearson correlation coefficient and the Manders’ coefficients—the Manders’ overlap coefficient (MOC) and the Manders’ correlation coefficient (MCC, which we used in this study). Both the Pearson and Manders’ coefficients are used to quantify the degree of colocalization between fluorophores, and the advantages of one over the other are actively debated [18]. Historically, MOC was introduced to overcome problems perceived with the Pearson correlation coefficient. The two coefficients are mathematically similar, differing in the use of either the absolute intensities (MOC) or the deviation from the mean (Pearson correlation coefficient). Both coefficients are independent of gain.

In our study, it was important to choose a colocalization analysis method that would not depend on cell thickness, shape, as well as the presence of a greater or lesser number of neighboring cells. On the other hand, the choice of the correlation coefficient can seriously affect the results [18,19]. In a number of cases, the accuracy of ROI selection is extremely important (in particular, in some cases, it is essential to choose a three-dimensional region of interest, 3D ROI) [20]. In addition, although for flat endothelial cells with thin lamellae, this choice of ROI does not seem to be so fundamental, it should be borne in mind that in endothelial cells growing in a dense confluent monolayer, thickness of the cytoplasm near the cell nuclei can significantly exceed the thickness of the lamellae.

Thus, taking the above into account, we acquired images using super-resolution microscopy, structured illumination microscopy. For the colocalization analysis, we selected cells that had contacts with neighboring cells; the analysis was performed discretely for specified regions within the same optical section, as well as for different optical sections for one region of interest. The degree of colocalization of β- and γ-actin was judged based on calculating the Manders’ coefficient [21], which is proportional to the amount of fluorescence of colocalizing pixels in each channel. The coefficient values range from 0 to 1, expressing the fraction of the intensity in a channel that is located in pixels where the intensity in another color channel is above zero (or a threshold value). Thus, the closer the Manders’ coefficient is to 1, the higher the colocalization of fluorescently colored structures of interest, while the Manders’ coefficient value close to 0 indicates the absence of colocalization.

### 2.2. Analysis of β- and γ-Actin Colocalization at the Cell Edge

**Figure 2:**
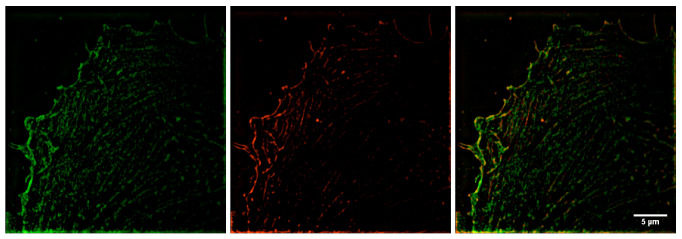
Immunofluorescent staining of β-(green) and γ-actin (red) in the basal region of HPAEC. In this optical section, β-actin apparently occupies a larger cell area than γ-actin.

**Figure 3:**
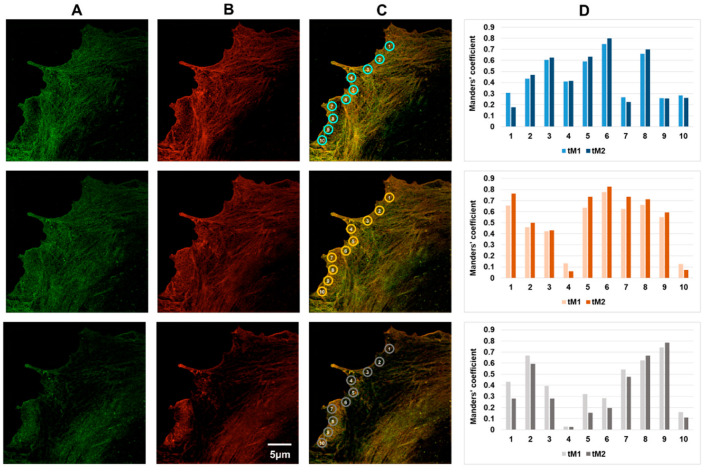
Colocalization of actin isoforms in the marginal regions of HPAEC. Vertical panel (**A**), immunofluorescent staining of β-actin; vertical panel (**B**), immunofluorescent staining of γ-actin; vertical panel (**C**), merged; vertical panel (**D**), three series of fluorescence intensity measurements in successive optical sections in the ROI defined by circles (marked in blue, orange and grey) at the cell edge. Measurements of the tM1 and tM2 threshold Manders’ coefficient in three adjacent optical sections are presented. Occasionally, in the adjacent sections for a given ROI, the Manders’ coefficients varied significantly. In general, lower coefficient values were observed at the stable cell edges.

**Figure 4:**
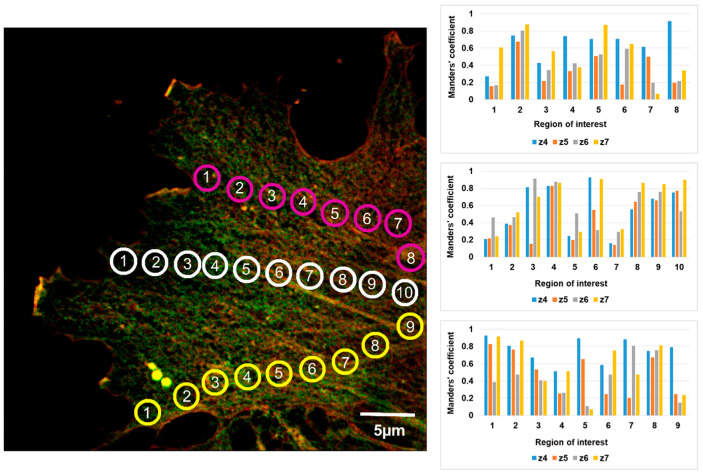
Colocalization of β- and γ-actin in different regions of the same HPAEC. Three measurement series were performed in ROI defined by circles (marked in magenta, white and yellow) and positioned in a line connecting the edge and center of the cell. Histograms present the respective tM1 Manders’ coefficient values. For each ROI in a series, the coefficient values are provided for four consecutive optical sections (two middlemost ones and two adjacent ones located above and below). In most cases, the tM1 coefficient values were noticeably below 1, indicating partial colocalization of β- and γ-actin. Minimal coefficient values were observed at the cell edge and adjacent to the cell nucleus (Series 1); at half way along the ROI-positioned line and at the cell edge (Series 2), or adjacent to the cell nucleus (Series 3).

**Figure 5:**
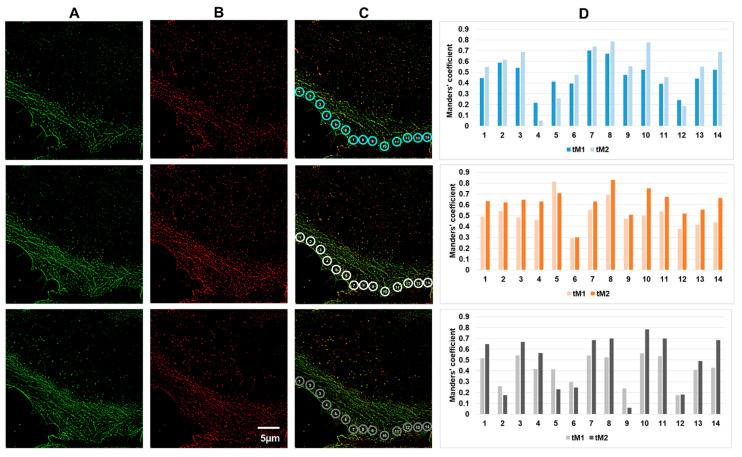
Colocalization of actin filaments in the marginal regions of EA.hy926 cells. Vertical panel (**A**), immunofluorescent staining of β-actin; vertical panel (**B**), immunofluorescent staining of γ-actin; vertical panel (**C**), merged, vertical panel (**D**), three series of fluorescence intensity measurements in successive optical sections in the ROI defined by circles (marked in blue, white and grey) at the cell edge. Measurements of the tM1 and tM2 threshold Manders’ coefficient in three adjacent optical sections are presented. Occasionally, in the adjacent sections for a given ROI, the Manders’ coefficients varied significantly.

Analysis of the whole z-stack showed that β-actin predominantly resided in the basal part of the cell, while in other parts of the cell, both β- and γ-actin structures were observed, i.e., actin isoforms were unevenly distributed within the cell volume (Figure 2, Figure 3, Figure 4 and Figure 5).

In vitro, the endothelial cell edge includes both relatively dynamic (active cell lamella) and relatively stable areas. In this study, we observed that at the cell edge, the level of β- and γ-actin colocalization varied often, depending on the activity/stability of this region.

### 2.3. Analysis of β- and γ-Actin Colocalization at Different Optical Sections of Artery and Vein Endothelial Cells

In a colocalization analysis in a stack of merged optical sections, the β- and γ-actin structures can look almost completely colocalized. However, a more thorough, layer-by-layer colocalization analysis in specific local areas of the cell contradicted the impression of complete colocalization of β- and γ-actin. It turned out that the colocalization coefficients can differ significantly not only in the neighboring areas of the endothelial cell lamella (Figure 3, ROI 1–10), but also at different depths within the selected area (i.e., in different optical sections) (Figure 3, panel C). The step (distance) between the adjacent optical sections was 0.12 μm.

We analyzed specific areas corresponding to functionally different zones of the active edge of endotheliocytes (Figure 3, areas 1–10) in different optical layers. We found that in different optical sections in different regions of the same cell, the colocalization coefficient can vary significantly, approximately from tM1 = 0.15 (low level of colocalization) to tM1 = 0.9 (high level of colocalization). Complete colocalization was not observed at any region.

The Manders’ coefficient was often low (less than 0.4) in the cell body remote both from the edge and from the nucleus, as well as in the region adjacent to the cell nucleus. On the other hand, in consecutive optical sections of the same region, the colocalization coefficients could vary considerably. A typical example (Figure 3) is series 1, ROI 5: tM1 = 0.507 for z5 optical section, tM1 = 0.525 for z6 optical section, and a prominent tM1 = 0.871 forz7 optical section. These data suggest that β- and γ-actin are not always colocalized and are spatially separated in the cytoplasm of HPAEC cells.

**Figure 6:**
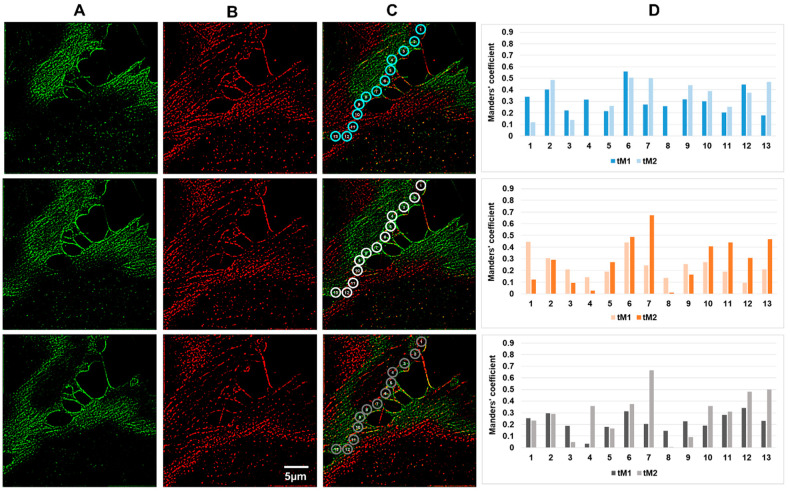
Analysis of colocalization of actin filaments in the marginal regions of EA.hy926 cell after 0.01 µM nocodazole treatment. Vertical panel (**A**), immunofluorescent staining of β-actin; vertical panel (**B**), immunofluorescent staining of γ-actin; vertical panel (**C**), merged, vertical panel (**D**), three series of fluorescence intensity measurements in successive optical sections in the ROI defined by circles (marked in blue, white and grey) at the cell edge. Measurements of the tM1 and tM2 threshold Manders’ coefficient in three adjacent optical sections are presented. Occasionally, in the adjacent sections for a given ROI, the Manders’ coefficients varied significantly.

Analysis of the β- and γ-actin colocalization in different parts of the same cell showed that these isoforms were incompletely colocalized (Figure 4). Obviously, as discussed above, variations in the β- and γ-actin colocalization coefficients between adjacent optical sections within a given ROI (see Figure 3 and Figure 4) and variations in the ROI number depending on the lamellae lengths (for example, these varied from 8 to 14 in Figure 3, Figure 4, Figure 5 and Figure 6) did not allow us to carry out a cell-to-cell comparison for an individual ROI. To evaluate statistical differences, we calculated and compared the average colocalization coefficients found in the cell lamella and in regions adjacent to the cell nucleus (Figure S1). For ROIs that were selected close to cell nuclei, the average Manders’ coefficient (tsM1 = 0.528 ± 0.042) was statistically higher than for the lamellae-located ROIs (tsM1 = 0.336 ± 0.042).

Next, we analyzed actin filaments in the EA.hy926 cell line. The analysis covered the marginal cell regions: free lamellae or zones of contact with a neighboring cell. In EA.hy926 cells, the Manders’ colocalization coefficient varied in a wide range from 0.05 to 0.86. The highest values were observed in border zones—near contacts with a neighboring cell next to a free edge (Figure 5, ROIs 7 and 8). In the contact-free edge, the colocalization coefficient varied significantly—in some regions of interest (Figure 5, ROI 2,4,5), the Manders’ coefficient changed dramatically depending on the optical section, probably due to the presence of cellular protrusions in these zones. In the region of cell-cell contacts, the coefficient values were more constant for a specific zone, but varied significantly from one ROI to another, probably indicating the maturity of local cellular contacts.

### 2.4. Segregation of β- and γ-Actin in the Nocodazole-Induced Endothelial Barrier Dysfunction

Since at the contact-free cell edge, the actin isoform colocalization coefficient varied most significantly, we investigated whether β- and γ-actin localization in this cytoplasm region would change under conditions other than normal.

The main function of endothelial cells is the barrier function. Under pathological conditions in vivo or under specific experimental interventions, barrier dysfunction may occur. The dysfunction is accompanied by cell cytoskeleton remodeling and activation of actomyosin contractility, resulting in cell contraction and intercellular gap formation. This ultimately leads to the destruction of intercellular VE-cadherin contacts and, as a consequence, to impaired endothelial permeability. Endothelial VE-cadherin contact disruption leads to the formation of active lamellae on the contact-free cell edges. Under experimental conditions that model the reversible endothelial dysfunction, it is this active lamella that, spreading out, ensures the cell shape restoration and reconstruction of intercellular contacts. Therefore, cell lamellae arising in the contact-free edges during barrier dysfunction development became the object of our interest. Based on our results obtained in HPAEC, we assumed that during the development of barrier dysfunction, the degree of β- and γ-actin colocalization may decrease even more significantly than observed under normal conditions.

Earlier, we established that depolymerization of endothelial cell peripheral microtubules is a trigger of barrier dysfunction [22,23]. Therefore, nocodazole, a classic microtubule disruptor, induces the endothelial barrier dysfunction in a dose-dependent manner [22,23]. Previously, we developed in vitro models of nocodazole-induced barrier impairment [24]; therefore, in the present study, we used nocodazole at low doses that caused the reversible barrier dysfunction in the EA.hy926 cell monolayer.

In EA.hy926 cells treated with nocodazole (0.01 µM), the colocalization coefficient of two actin isoforms did not exceed 0.68 and, even, in some ROI, decreased to 0 (an indication of the absence of β- and γ-actin colocalization). In the cell-cell contact zone, the colocalization coefficients were usually higher than in the contact-free lamellae but still did not exceed 0.5 (Figure 6).

Many authors suggested there are differences in functions of β- and γ-actin [1,8,10,25,26]. In particular, β-actin functions as an essential regulator of gut barrier integrity in vivo and plays a tissue protective role during mucosal injury and inflammation [27]. In normal epithelial cells, cytoplasmic actin isoforms are segregated during anaphase and telophase, playing different roles in the mitotic cell division [1,28]. Loss of gamma-cytoplasmic actin triggers myofibroblast transition of epithelial cells [29]. Downregulation of cytoplasmic actin isoforms alters the phenotype and karyotype of breast cancer cells;β-actin depletion leads to the progression of chromosomal instability with endoreduplication and aneuploidy increase, but γ-actin downregulation results in chromosome stability, reduced polyploidy, and aneuploidy, the reducing percentage of mitotic carcinoma cells [30]. Depletion of each cytoplasmic actin leads to impaired proliferation/cell cycle of carcinoma cells [31,32] and two cytoplasmic actin isoforms play different roles in neoplastic cell transformation. In cancer cells, β-actin acts as a tumor suppressor, whereas γ-cytoplasmic actin enhances malignant features of tumor cells [31]. Overexpression of β- or γ-actin leads to remodeling of actin structure, increased cell migration and invasion capacities, but velocity of migration is higher in cells with overexpressed γ-actin [33]. Both isoforms are found in invadopodia of mesenchymal cancer cells [34] but modulation of β-actin expression level (overexpression, silencing, or knockout) differs from γ-actin and leads to opposite results [26].

Differences in the colocalization degree of two actin isoforms may be explained by their functional differences, including the fact that they interact differently with microtubules (similar to that described for epithelial cells [35]), as well as other components of the cell contractile complex. Moreover, in in vitro experiments, non-sarcomeric myosin-7A preferentially interacts with γ-actin in comparison with β-actin filaments, though the latter predominantly activates non-muscle myosin 2C1 [9]. We speculate that for endothelial cells, spatial separation of cytoplasmic γ- and β-actin is a fundamental functional property. That is, there are several local endothelial cell sites with different functional activity, including interaction points with linker proteins that connect actin structures with microtubules, intermediate filaments, and VE-cadherin cell-cell contacts. This assumption is further supported by our experiments demonstrating that reversible endothelial barrier dysfunction is accompanied by a significant decrease up to the complete absence of β- and γ-actin colocalization.

## 3. Materials and Methods

### 3.1. Cell Cultures and Treatment

Human pulmonary artery endothelial cells (HPAEC), primary cells isolated from the human pulmonary artery, were used for analysis. HPAEC were obtained from Clonetics BioWhittaker Inc. (Frederick, MD, USA). Cells were maintained in EGM-2 medium (Clonetics, BioWhittaker, Inc., Frederick, MD, USA) at 37 °C in atmosphere of 5% CO_2_. We also used EA.hy926 vein endothelial cells. Originally, EA.hy926 cells were obtained by merging primary endotheliocytes isolated from human umbilical vein and cells of the thioguanine-resistant clone A549 (human lung carcinoma cells). Cells were grown at 37 °C and 5% CO_2_ in DMEM (Dulbecco’s Modified Eagle Media) (PanEco, Moscow, Russia) with the addition of 10% fetal bovine serum (FBS) (Hyclone, Logan, UT, USA), 2 mM glutamine (Hyclone, Logan, UT, USA) and 50 units/mL of penicillin-streptomycin (PanEco, Moscow, Russia). Experiments were performed in cultures at 6–10th passages. For modeling the endothelial barrier disruption, endothelial cells were stimulated with 0.01 µM nocodazole (Sigma, St. Louis, MO, USA). Nocodazole stock solutions were prepared in DMSO. Final concentrations of DMSO in the cell medium did not exceed 0.1%.

### 3.2. Immunofluorescence

For immunolabeling the actin isoforms, cells were grown on glass coverslips and fixed in 1% paraformaldehyde solution (Sigma, St. Louis, MO, USA) in DMEM medium (PanEco, Moscow, Russia) containing HEPES buffer (Sigma, St. Louis, MO, USA) for 15 min, then rinsed with PBS and fixed for an additional 5 min with methanol at −20 °C. Before fixing, some coverslips were incubated with nocodazole at a concentration of 0.01 μM for 30 min at 37 °C and 5% CO_2_.

Actin filaments were stained with murine monoclonal antibodies against cytoplasmic β- or γ-actin [1]. Anti-mouse antibodies conjugated with Alexa 488 or Alexa 561 fluorescent dyes (Molecular Probes, Eugene, OR, USA) were used as secondary antibodies.

The samples were embedded in Moviol and examined using an N-SIM microscopic system ((Nikon Instech Co., Tokyo, Japan) with an immersion objective 100×/1.49 NA, excitation laser wavelengths of 488 nm and 561 nm. Image stacks (with a *z*-axis step of 0.12 μm) were acquired with an EMCCD camera (iXon 897, Andor, effective pixel size 60 nm) in the 3D-SIM mode. Serial optical sections of the same cell, taken in the wide field mode, were processed using the AutoQuant blind deconvolution algorithm. Image acquisition and SIM reconstruction were performed using the NIS-Elements 4.2 software (Nikon Instech Co., Tokyo, Japan).

### 3.3. Correlation Analysis of Cytoplasmic Actin Isoforms Distribution

**Figure 7:**
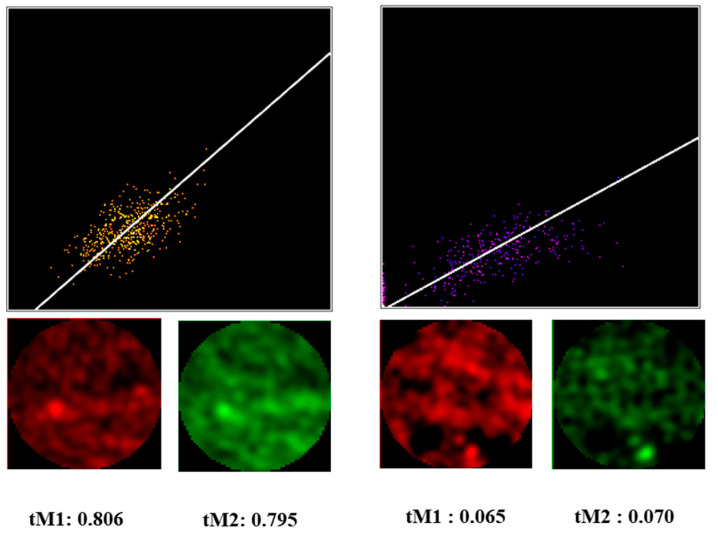
Colocalization of β- and γ-actin in the endothelial cell lamella: the Manders’ coefficient. Scatterplots of pixel intensity correlation reflect regions with high (**left**) and low (**right**) colocalization coefficients.

Colocalization analysis of both wide-field and SIM images was performed using the Coloc2 plugin of the ImageJ 1.52 m software (National Institutes of Health, Bethesda, MD, USA). For the correlation analysis, circular ROIs of 2 µm diameter were selected in individual optical sections (Figure 7). The background was subtracted before ROI selection. ROIs were positioned both along a line running from the cell center to its edge and in the edge areas of the cell where we positioned ROIs in functionally differing areas, both stable or active edge regions. For each ROI, the colocalization analysis was performed in three consecutive optical sections for which threshold Manders’ coefficients were calculated with automatic threshold settings defined by Costes regression approach. Since the thickness of endotheliocyte’s lamella is about 0.5 µm, an image series usually consists of 3–4 sections. For the analysis, we selected the middlemost optical section and two adjacent optical sections lying above and below it. To find out whether β- and γ-actin are colocalized in a given region, we used the Coloc2 plugin function of calculating the M1 and M2 Manders’ coefficients (i.e., separately for two channels). The modified tM1 and tM2 coefficients (threshold Manders’ coefficients) were calculated, for which the ImageJ software threshold values were used (Figure 7).

For each type of analysis, data from three independent experiments were used; in total, 10 cells were analyzed for each region of interest. To evaluate statistical differences, additionally, the average colocalization coefficients in the cell lamella and in regions adjacent to the cell nucleus were calculated (Figure S1). The average Manders’ coefficients were compared by Mann–Whitney U Statistic test.

## 4. Conclusions

This study convincingly demonstrates that in HPAEC, β and γ-actin are partially colocalized in certain regions of the endothelial cytoplasm. The degree of colocalization of β- and γ-actin varies depending on the cell region, i.e., there are regions with both a low degree of colocalization and a high one.

Disturbances in endothelial cell barrier regulation are critically dependent upon re-arrangements of endothelial cell actin cytoskeleton. A functional assay with experimentally-induced endothelial cell barrier dysfunction demonstrated significant growth of β- and γ-actin segregation accompanying cell lamella activation.

Based on our findings, we conclude that an increase in the β- and γ-actin segregation in the nocodazole-induced endothelial barrier dysfunction may reflect different functional roles of two cytoplasmic actin isoforms in the functional activity of endothelial cells.

The correlation coefficient can vary significantly, even in adjacent optical sections within a given region. This may indicate the local functional activity of individual β- and γ-actin cytoskeleton structures in the endotheliocyte lamella. In the vicinity of forming contacts, where the cell edge remains rather thin, the correlation coefficient of the isoforms is relatively constant and varies in the range of 0.48–0.67, which reflects the involvement of both actin isoforms in the process of intercellular VE-cadherin contact formation and maintaining the endothelial barrier. At the same time, in contact-free cell edges, the correlation coefficient can vary in a much wider range, from 0.18 to 0.90, which is apparently due to dynamism of this region. The alternation of functionally different (active and stable) zones in the leading edge of endothelial cells reveals the different involvement of β- and γ-actin structures.

## Data Availability

The data presented in this study are available in https://pubmed.ncbi.nlm.nih.gov/.

## Supplementary Materials

The following supporting information can be downloaded at: https://www.mdpi.com/article/10.3390/biomedicines10123194/s1, Figure S1: Manders’ coefficients were calculated for β- and γ-actin structures colocalization analysis in the zone of free lamellae and areas adjacent to the cell nucleus of HPAEC.

## References

[1] Dugina V., Zwaenepoel I., Gabbiani G., Clement S., Chaponnier C. (2009). β- and γ-cytoplasmic actins display distinct distribution and functional diversity. J. Cell Sci..

[2] Latham S.L., Chaponnier C., Dugina V., Couraud P.-O., Grau G.E.R., Combes V. (2013). Cooperation between β- and γ-cytoplasmic actins in the mechanical regulation of endothelial microparticle formation. FASEB J..

[3] Shakhov A.S., Verin A.D., Alieva I.B. (2014). Reorganization of endothelial cells cytoskeleton during formation of functional monolayer in vitro. Cell Tissue Biol..

[4] Shakhov A.S., Dugina V.B., Alieva I.B. (2015). Reorganization of actin and microtubule systems in human vein endothelial cells during intercellular contact formation. Cell Tissue Biol..

[5] Dominguez R., Holmes K.C. (2011). Actin structure and function. Annu. Rev. Biophys..

[6] Rottner K., Faix J., Bogdan S., Linder S., Kerkhoff E. (2017). Actin assembly mechanisms at a glance. J. Cell Sci..

[7] Vandekerckhove J., Weber K. (1978). At least six different actins are expressed in a higher mammal: An analysis based on the amino acid sequence of the amino-terminal tryptic peptide. J. Mol. Biol..

[8] Ampe C., Van Troys M. (2016). Mammalian Actins: Isoform-Specific Functions and Diseases. Handbook of Experimental Pharmacology.

[9] Müller M., Diensthuber R.P., Chizhov I., Claus P., Heissler S.M., Preller M., Taft M.H., Manstein D.J. (2013). Distinct Functional Interactions between Actin Isoforms and Nonsarcomeric Myosins. PLoS ONE.

[10] Pasquier E., Tuset M.-P., Sinnappan S., Carnell M., Macmillan A., Kavallaris M. (2015). γ-Actin plays a key role in endothelial cell motility and neovessel maintenance. Vasc. Cell.

[11] Peñarrubia P.G., Ruiz X.F., Gálvez J. (2005). Quantitative analysis of the factors that affect the determination of colocalization coefficients in dual-color confocal images. IEEE Trans. Image Process..

[12] Oheim M., Li D., Shorte S.L., Frischknecht F. (2007). Quantitative Colocalisation Imaging: Concepts, Measurements, and Pitfalls. Quantitative Colocalisation Imaging: Concepts, Measurements, and Pitfalls.

[13] Nakamura K., Watakabe A., Hioki H., Fujiyama F., Tanaka Y., Yamamori T., Kaneko T. (2007). Transiently increased colocalization of vesicular glutamate transporters 1 and 2 at single axon terminals during postnatal development of mouse neocortex: A quantitative analysis with correlation coefficient. Eur. J. Neurosci..

[14] Zinchuk V., Zinchuk O., Okada T. (2007). Quantitative colocalization analysis of multicolor confocal immunofluorescence microscopy images: Pushing pixels to explore biological phenomena. Acta Histochem. Cytochem..

[15] Adler J., Pagakis S.N., Parmryd I. (2008). Replicate-based noise corrected correlation for accurate measurements of colocalization. J. Microsc..

[16] French A.P., Mills S., Swarup R., Bennett M.J., Pridmore T.P. (2008). Colocalization of fluorescent markers in confocal microscope images of plant cells. Nat. Protoc..

[17] Zinchuk V., Grossenbacher-Zinchuk O. (2009). Recent advances in quantitative colocalization analysis: Focus on neuroscience. Prog. Histochem. Cytochem..

[18] Adler J., Parmryd I. (2010). Quantifying colocalization by correlation: The pearson correlation coefficient is superior to the Mander’s overlap coefficient. Cytom. Part A.

[19] Theart R.P., Loos B., Niesler T.R. (2017). Virtual reality assisted microscopy data visualization and colocalization analysis. BMC Bioinform..

[20] Theart R.P., Loos B., Powrie Y.S.L., Niesler T.R. (2018). Improved region of interest selection and colocalization analysis in three-dimensional fluorescence microscopy samples using virtual reality. PLoS ONE.

[21] Manders E.M.M., Verbeek F.J., Aten J.A. (1993). Measurement of co-localization of objects in dual-colour confocal images. J. Microsc..

[22] Birukova A.A., Smurova K., Birukov K.G., Usatyuk P., Liu F., Kaibuchi K., Ricks-Cord A., Natarajan V., Alieva I., Garcia J.G.N. (2004). Microtubule disassembly induces cytoskeletal remodeling and lung vascular barrier dysfunction: Role of Rho-dependent mechanisms. J. Cell Physiol..

[23] Birukova A.A., Birukov K.G., Smurova K., Adyshev D., Kaibuchi K., Alieva I., Garcia J.G.N., Verin A.D. (2004). Novel role of microtubules in thrombin-induced endothelial barrier dysfunction. FASEB J..

[24] Alieva I.B., Zemskov E.A., Smurova K.M., Kaverina I.N., Verin A.D. (2013). The leading role of microtubules in endothelial barrier dysfunction: Disassembly of peripheral microtubules leaves behind the cytoskeletal reorganization. J. Cell Biochem..

[25] Baranwal S., Naydenov N.G., Harris G., Dugina V., Morgan K.G., Chaponnier C., Ivanov A.I. (2012). Nonredundant roles of cytoplasmic β- and γ-actin isoforms in regulation of epithelial apical junctions. Mol. Biol. Cell.

[26] Simiczyjew A., Pietraszek-Gremplewicz K., Mazur A.J., Nowak D. (2017). Are non-muscle actin isoforms functionally equivalent?. Histol. Histopathol..

[27] Lechuga S., Naydenov N.G., Feygin A., Cruise M., Ervasti J.M., Ivanov A.I. (2020). Loss of β-Cytoplasmic Actin in the Intestinal Epithelium Increases Gut Barrier Permeability in vivo and Exaggerates the Severity of Experimental Colitis. Front. Cell Dev. Biol..

[28] Shagieva G.S., Alieva I.B., Chaponnier C., Dugina V.B. (2020). Divergent Impact of Actin Isoforms on Division of Epithelial Cells. Biochemistry.

[29] Lechuga S., Baranwal S., Li C., Naydenov N.G., Kuemmerle J.F., Dugina V., Chaponnier C., Ivanov A.I. (2014). Loss of γ-cytoplasmic actin triggers myofibroblast transition of human epithelial cells. Mol. Biol. Cell.

[30] Dugina V.B., Shagieva G.S., Shakhov A.S., Alieva I.B. (2021). The cytoplasmic actins in the regulation of endothelial cell function. Int. J. Mol. Sci..

[31] Dugina V., Khromova N., Rybko V., Blizniukov O., Shagieva G., Chaponnier C., Kopnin B., Kopnin P. (2015). Tumor promotion by γ and suppression by β non-muscle actin isoforms. Oncotarget.

[32] Dugina V., Shagieva G., Khromova N., Kopnin P. (2018). Divergent impact of actin isoforms on cell cycle regulation. Cell Cycle.

[33] Simiczyjew A., Mazur A.J., Popow-Woźniak A., Malicka-Błaszkiewicz M., Nowak D. (2014). Effect of overexpression of β- and γ-actin isoforms on actin cytoskeleton organization and migration of human colon cancer cells. Histochem. Cell Biol..

[34] Simiczyjew A., Mazur A.J., Ampe C., Malicka-Błaszkiewicz M., van Troys M., Nowak D. (2015). Active invadopodia of mesenchymally migrating cancer cells contain both β and γ cytoplasmic actin isoforms. Exp. Cell Res..

[35] Dugina V., Alieva I., Khromova N., Kireev I., Gunning P.W., Kopnin P. (2016). Interaction of microtubules with the actin cytoskeleton via cross-talk of EB1-containing +TIPs and γ-actin in epithelial cells. Oncotarget.

