# Supplementary material for: Colocalization Analysis of Cytoplasmic Actin Isoforms Distribution in Endothelial Cells
Source: Biomedicines. 2022 Dec 9;10(12):3194. doi: 10.3390/biomedicines10123194 (PMC9775052; doi:10.3390/biomedicines10123194)
Supplement: Supplementary file 1 [file biomedicines-10-03194-s001.zip › biomedicines-1975665-supplementary.pdf]

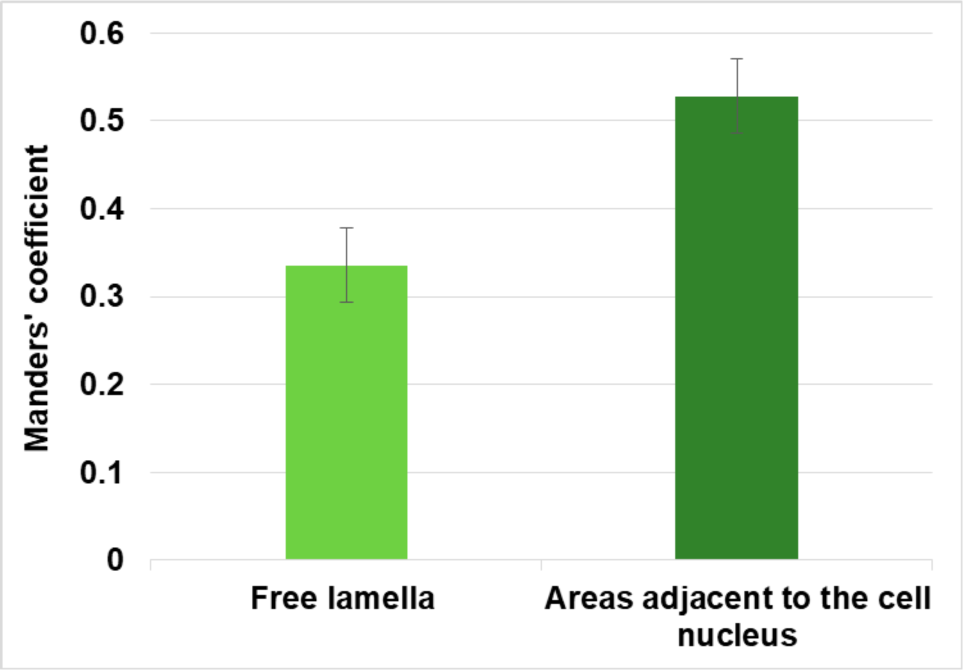

Figure S1. Manders’ coefficients were calculated for β- and γ-actin structures colocalization analysis in the zone of free lamellae and areas adjacent to the cell nucleus of HPAEC. We compared two groups of ROI in different cell areas. The average Manders’ (tM1=0.528±0.042) coefficient in ROI that are located close to the nucleus was statistically higher than in ROI of the free lamella (tM1=0.336±0.042). The difference in the median values between the two groups was greater than would be expected by chance (statistically significant difference, P = 0.008).
